# A Recurrent Platinum Refractory Ovarian Cancer Patient With a Partial Response After RRx-001 Resensitization to Platinum Doublet

**DOI:** 10.1177/2324709618760080

**Published:** 2018-03-07

**Authors:** Hope Cottrill, Stephanie Cason, Scott Caroen, Bryan Oronsky, Elvis Donaldson

**Affiliations:** 1 Baptist Health, Lexington, KY, USA; 2 EpicentRx, San Diego, CA, USA

**Keywords:** epithelial ovarian cancer, platinum doublet, RRx-001

## Abstract

This case report describes the clinical course of a heavily pretreated patient enrolled on the Phase II QUADRUPLE THREAT clinical trial (NCT02489903) with recurrent metastatic epithelial ovarian cancer that was previously treated with a platinum doublet and that benefited from another reintroduction of a platinum doublet after a brief priming period with RRx-001. Platinum-taxane combination is the initial treatment of choice in ovarian cancer and the “go-to regimen” for patients with recurrent, platinum-sensitive disease. However, platinum resistance is an inevitable and uniformly fatal development. One of the “Holy Grails” in ovarian cancer, besides early detection, is to reverse this resistance and retreat with a platinum doublet regimen. Because of the correlation between therapeutic resistance and poor clinical outcomes, this case, which potentially demonstrates a new avenue of anticancer development in epithelial ovarian cancer provided it is repeatable with other ovarian patients, was felt to merit a report.

## Introduction

The historical and current cornerstone of treatment for advanced ovarian cancer is primary debulking surgery followed by platinum-based chemotherapy. Unfortunately, the high initial response rates achieved with platinum therapy are not durable, as chemosensitivity typically gives way to a broad chemo cross-resistance that encompasses not just platinum but also multiple drugs to which patients were never previously exposed.^1^ The dismal 5-year survival rate of 17%^2^ in stage 4 epithelial ovarian cancer (EOC) is primarily attributable to the emergence of resistance, which has a genetic and epigenetic basis.^3^ However, in contrast to the permanence of genetic defects, epigenetic mechanisms are dynamic and potentially reversible with epigenetic therapies, which accounts for their appeal when used as pretreatment “primers” to prevent or reverse nonresponsiveness to chemotherapy. Previous experience with RRx-001, an experimental tumor-associated macrophage and neutrophil repolarizing agent with epigenetic and vascular normalization properties, has demonstrated resensitization to platinum in other tumor types, such as colorectal, small cell lung cancer (SCLC), and non-SCLC (NSCLC).^4^ This case report is the first to describe RRx-001-mediated resensitization to platinum in a heavily pretreated metastatic EOC patient on the Phase II QUADRUPLE THREAT clinical trial (NCT02489903). This clinical trial investigates the effectiveness of RRx-001 to sensitize to retreatment with platinum doublet therapy in patients of 4 different cancer histologies (SCLC, NSCLC, neuroendocrine tumors, and ovarian cancer). The clinical trial was approved by the relevant institutional review boards.

## Case Report

This 49-year-old Caucasian female, G4P3, was diagnosed in 2011 with International Federation of Gynecology and Obstetrics stage IIIC BRCA-mutation negative papillary serous adenocarcinoma metastatic to liver and peritoneum and underwent tumor-debulking surgery. At the time of diagnosis, her CA-125 was approximately 3500. She subsequently received 6 lines of systemic therapy as detailed below:

*Regimen 1*: Intravenous injection/intraperitoneal injection cisplatin and taxol × 6 cycles from January 2012 to May 2012, which led to remission of disease for around 1 year.
*Regimen 2*: Olaparib × 13 cycles with progression of disease in March 24, 2014.
*Regimen 3*: GYN44 (antibody conjugate) × 6 with carboplatin × 4 cycles, which was stopped in January 2015 due to neuropathy and anemia.
*Regimen 4*: Heat treatment × 4, which was completed in August 2015.
*Regimen 5*: Cediranib + Medi4736 (checkpoint inhibitor and anti-PDL1) from September 2015 to March 2016 with progression of disease.
*Regimen 6*: Doxil + carboplatin for 8 cycles with discontinuation due to progression and neuropathy.

**Figure 1.**
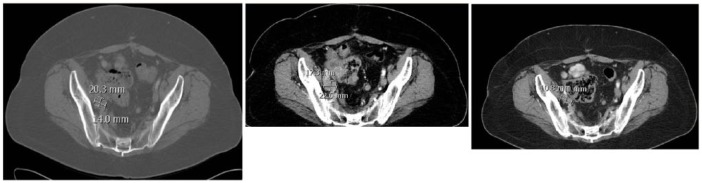
Computed tomography scans, from left to right, showing a pelvic target lesion (1) at baseline measuring 20.3 mm largest diameter, (2) after 1 month of RRx-001 treatment measuring 22.6 largest diameter, and (3) after 2 cycles of carboplatin-taxane measuring 10.8 mm largest diameter.

At the time of the patient’s enrollment on the QUADRUPLE THREAT trial on February 2, 2017, her ECOG (Eastern Cooperative Oncology Group) performance status was 0 despite recent rapid weight loss and increased use of opioids for intractable abdominal cancer pain. The patient received her first intravenous infusion of RRx-001, dosed once weekly, on March 7, 2017. At that time (baseline) her CA-125 was 1200. During RRx-001 treatment, she experienced no drug-related toxicities; however, her chronic abdominal pain slightly worsened, which required increasing doses of opioid agonists, and her CA-125 started to steadily increase. By the time she was imaged 4 weeks later on April 4, 2017, her CA-125 had reached 4000. While her scan revealed grossly stable disease, the combination of slight clinical worsening and increasing levels of CA-125 prompted a switch to carboplatin-taxane on April 6, 2017. After Cycle 1 of carbo-taxane her CA-125 decreased to 1600. At the end of Cycle 2 on May 23, 2017, despite detection of an asymptomatic pulmonary embolism for which the factor Xa inhibitor, apaxiban (Eliquis) was started, the patient was exhibiting radiographic, biochemical, and symptomatic improvement: target lesions were significantly smaller (computed tomography images shown in Figure 1), resulting in a partial response; her CA-125 was 830 (lower than baseline of 1200); and her abdominal pain had entirely disappeared, resulting in voluntary discontinuation from all opioid medications.

The patient completed 6 cycles of platinum doublet and at the time of progression she was restarted on RRx-001 through a compassionate use process.

## Discussion

A major—if not the major—problem in ovarian cancer is chemoresistance, and epigenetic changes are increasingly implicated. For clarity, in this context, the term “epigenetic changes” refers to transcriptional repression or a “gene-off” state mediated by DNA methylation and histone modifications; the potential of epigenetic therapies to de-repress epigenetically silenced genes^5^ and restore platinum sensitivity in resistant recurrent EOC was first demonstrated in 2 clinical trials: the initial one in 2011 was a 30 patient trial by Fu et al^6^ with concomitant azacitidine and carboplatin, and the second in 2012 was a 17 patient trial by Matei et al^7^ with low-dose decitabine administered prior to carboplatin, where the objective response rates of 35% and 22%, respectively, exceeded the historical objective response rate of <10%.^8^ That these encouraging results from 2011 and 2012 have not led to widespread adoption of epigenetic strategies in ovarian cancer is presumably because of the toxicity rates of the myelosuppressive DNA demethylators, decitabine and azacitidine, in combination with a platinum. However, in contrast to decitabine and azacitidine, RRx-001 is a non-myelosuppressive epigenetic inhibitor, vascular normalizer, and immunotherapeutic^9^ that may even attenuate platinum-induced toxicities.^10^

Despite an array of clinically approved or investigational agents available or potentially available in ovarian cancer including PARP inhibitors such as olaparib and niraparib, immunotherapies such as pembrolizumab and nivolumab, antibody drug conjugate agents such as DMUC5754A and IMGN853, and anti-angiogenesis agents such as bevacizumab and cediranib, the armamentarium is far from complete: EOC still remains the most lethal gynecologic malignancy, and as a result, additional and alternative therapeutic strategies to improve largely stagnant outcomes are urgently needed.

This case report is the first to describe resensitization to carboplatin and taxane in a heavily pretreated patient in her seventh line of therapy after a brief priming period with RRx-001. Biopsy of the patient’s tumors may help establish the specific epigenetic (or other) mechanism(s), for example, de-repression of silenced tumor suppressor genes and/or activation of the adaptive immune system by which resensitization to carboplatin occurred, the better to develop a predictive biomarker for prospectively determining which patients are most likely to benefit from rechallenge with platinum doublet after RRx-001 therapy. Rechallenge in platinum-resistant patients is theoretically not without risk, since the carboplatin-paclitaxel regimen, in particular, is associated with neuropathy and myelosuppression, which argues for the use of a predictive biomarker; however, even without such a biomarker, RRx-001, as a putative broad-spectrum dual chemoprotector and chemosensitizer,^11^ may generally improve the therapeutic index of the doublet for unselected patients because of a better antitumor effect with lower toxicity. A pivotal or pivotal trials is required to confirm these hypotheses and to determine whether RRx-001 will be eventually added to the ovarian cancer armamentarium not only to reverse resistance in late recurrence but also potentially to prevent it in early-stage disease.
